# Molybdenum Disulfide-Integrated Iron Organic Framework Hybrid Nanozyme-Based Aptasensor for Colorimetric Detection of Exosomes

**DOI:** 10.3390/bios13080800

**Published:** 2023-08-09

**Authors:** Chao Li, Zichao Guo, Sisi Pu, Chaohui Zhou, Xi Cheng, Ren Zhao, Nengqin Jia

**Affiliations:** 1 The Education Ministry Key Lab of Resource Chemistry, Joint International Research Laboratory of Resource Chemistry, Ministry of Education, Shanghai Frontiers Science Center of Biomimetic Catalysis and Shanghai Key Laboratory of Rare Earth Functional Materials, College of Chemistry and Materials Science, Shanghai Normal University, Shanghai 200234, China; 2 Department of General Surgery, Ruijin Hospital, Shanghai Jiao Tong University School of Medicine, Shanghai 200025, China

**Keywords:** exosome, aptamer, nanozyme, colorimetric assay, metal organic framework

## Abstract

Tumor-derived exosomes are considered as a potential marker in liquid biopsy for malignant tumor screening. The development of a sensitive, specific, rapid, and cost-effective detection strategy for tumor-derived exosomes is still a challenge. Herein, a visualized and easy detection method for exosomes was established based on a molybdenum disulfide nanoflower decorated iron organic framework (MoS_2_-MIL-101(Fe)) hybrid nanozyme-based CD63 aptamer sensor. The CD63 aptamer, which can specifically recognize and capture tumor-derived exosomes, enhanced the peroxidase activity of the hybrid nanozyme and helped to catalyze the 3,3′,5,5′-tetramethylbenzidine (TMB)-H_2_O_2_ system to generate a stronger colorimetric signal, with its surface modification on the hybrid nanozyme. With the existence of exosomes, CD63 aptamer recognized and adsorbed them on the surface of the nanozyme, which rescued the enhanced peroxidase activity of the aptamer-modified nanozyme, resulting in a deep-to-moderate color change in the TMB-H_2_O_2_ system where the change is visible and can be monitored with ultraviolet-visible spectroscopy. In the context of optimal circumstances, the linear range of this exosome detection method is measured to be 1.6 × 10^4^ to 1.6 × 10^6^ particles/μL with a limit of detection as 3.37 × 10^3^ particles/μL. Generally, a simple and accessible approach to exosome detection is constructed, and a nanozyme-based colorimetric aptamer sensor is proposed, which sheds light on novel oncological biomarker measurements in the field of biosensors.

## 1. Introduction

Malignant tumor is one of the main causes of lethal casualty worldwide, presenting with escalating morbidity among the developing and underdeveloped countries. Besides effective therapy, early screening also plays an important role in the fight against malignant tumors, which raises a huge demand for a large volume of precise and repeatable detection [1,2]. However, the common screening strategies for malignant tumors, including positron emission tomography, magnetic resonance imaging, computed tomography, X-ray, and endoscopy, are not suitable for large-volume screening and are not accessible in primary clinics. In addition to radiological imaging examinations, tumoral biomarkers detections in liquid samples are widely used in tumor screening, diagnosis, and follow-up [3]. Although a variety of novel potential biomarkers have been identified for different cancers, methodological and technical obstacles still exist in terms of biomarker detection and limit its further applications, such as low concentration or poor stability of biomarkers in human body fluid samples, which caused attention in sensitive and stable biomarker-detecting techniques [4].

As first observed in sheep reticulocytes in the 1980s [5], exosomes are endosomal-originated extracellular vesicles with an average diameter of 100 nm. Exosomes are discovered in various human body fluids, carrying lipids, proteins, nucleic acids, and other metabolites, which were suggested to have a role in intercellular communications that maintains gene transcription and translation, cell proliferation, metabolic reprogramming, angiogenesis, immune response, cellular differentiation, and migration [6]. Emerging studies have shown that exosomes can be used as a novel, promising, and reliable biomarker for cancer screening in the field of breast cancer and pancreatic cancer [7,8]. Increasing evidence has shown that tumor-derived exosomes are involved in carcinogenesis, tumor progression, and chemoresistance by regulating intercellular communications and reshaping the tumor microenvironment. As a flourishing research topic, exosome detection is not only applicable for cancer screening, diagnosis, and follow-up but also crucial for future in-depth exploration of the exact physiological and biochemical characteristics of tumor-derived exosomes.

As a unique class of single-stranded oligonucleotides [9], aptamers fold into specific tertiary structures to serve as recognition ligands that can attach to their targets with high affinity and specificity. With its programmable, modifiable, and engineerable designs, aptamer sensors, have added a novel flourished dimension to the field of liquid biopsy. Particularly, with their characteristics of synthetic and stability, aptamer sensors show their great accessibility and economic efficiency over traditional antibodies. Functioning as recognition ligands, there are emerging studies reporting that aptamer sensors were applied for exosome detection [10,11].

At present, well-recognized methods, such as Western blot [12], nanoparticle tracking analysis (NTA) [13,14], and flow cytometry have been introduced for the identification and quantification of exosomes. However, obstacles raised by technical limitations still hinder the widespread application of exosome detection. Demands for customized instruments, specialized software, and relevant reagents limit their usability for clinical situations, especially in primary clinics. Since 2017 [15], aptamers have become widely used ligands for exosome detector construction. In terms of the signal transduction methods, aptamers sensors can be divided into the following categories: fluorescence, colorimetric, electrochemical, and luminescent, etc. The colorimetric assay is an absorbance-based quantification method that can be easily implemented without exquisite instruments, which takes the advantages of accessibility, affordability, easy operation and quick results compared to the other assays and has been used for exosome detection [15].

The rapid development of enzymes has boosted the explorations of various novel biosensors. As promising candidates for natural biological enzymes, nanozymes have attracted increasing attention because of their characteristics of stability, reasonable price, and batch production with uncompromised catalytic efficiency. Ever since Fe_3_O_4_ nanoparticles [16,17,18] were reported to have a peroxidase-like (POD-like) catalytic activity, more and more nanomaterials have been suggested to have different catalytic activities, including carbon nanohybrid materials [19,20], inorganic nanomaterials [21,22] and metal-organic skeleton materials [23,24]. Recently, the peroxidase-based colorimetric assays have been proposed for exosomes detection [25,26]. As a well-recognized colorimetric method, different peroxidase catalytic activities can oxidize substrates including 3,3’,5,5’-tetramethylbenzidine (TMB) with the existence of hydrogen peroxide (H_2_O_2_), exhibiting different absorbance, which can be measured quantitatively.

The metal-organic framework (MOF), as a new kind of self-assembled three-dimensional orderly coordinated polymer, is constructed by coordination bonds of organic linkers and metal ions/clusters [27], presenting the characteristics of tremendous specific surface area and large porosity. Despite these merits, owing to the presence of organic ligands and their relatively high molecular weight, MOF-based nanozymes still need improvement in the highly accessible active sites and catalytic efficiency [28,29,30]. Functionalized MOF-based nanozymes, and nanozymes with various exposed active units are considered very promising solutions, among which the MOF-based hybrid nanozymes constructed by hybridization regulation strategy could considerably enhance the catalytic activity of the nanozyme due to their synergistic effects of hybrid materials beneficial for the improvement of dispersion, conductivity, and specific surface area.

In terms of molybdenum disulfide (MoS_2_), a transition metal dihalide compound, presented as a two-dimensional, flake, and graphene-like structure, exhibits abundant catalytic-active edges and advanced specific surface area [31]. Recently, MoS_2_ nanosheets have been reported as a promising mimic of peroxidase [32]. To date, in several functional materials [33,34], MoS_2_ has been adopted as the subunit to mimic natural enzymes and achieve enhanced catalytic activity. Therefore, MoS_2_ is an ideal alternative for the construction of MOF-based nanozyme.

In this work, a visible exosome detection technique was developed based on an artificial, mimetic nanozyme of peroxidase, the MoS_2_-MIL-101(Fe) hybrid nanozyme was constructed and was proved to possess superior peroxidase enzymatic activity. The configuration of MIL-101(Fe) provided MoS_2_ with a large specific surface area, which is conducive to the absorption of substrates on the exterior of the hybrid nanozyme. Additionally, modified on the exterior of the hybrid nanozyme via electrostatic interaction, the CD63 aptamers not only specifically recognize and capture exosomes but also enhances the affinity of the hybrid nanozyme to its substrates and further improve its catalytic activity with the aptamer’s single-strand DNA configuration [35,36]. Generally, with these synergistic effects, the MoS_2_-MIL-101(Fe) hybrid nanozyme-based aptamer sensor is assumed to have an ideal detection limit. And a multi-purpose design of the nanozyme-based colorimetric aptamer sensor is proposed.

## 2. Experimental Section

### 2.1. Chemicals, Reagents and Cell Lines

3,3′,5,5′-tetramethylbenzidine (TMB) and terephthalic acid were purchased from Energy Chemical (Shanghai, China). Ammonium thiomolybdate ((NH_4_)_2_MoS_4_), ferric chloride hexahydrate (FeCl_3_·6H_2_O), ethanol (CH_3_CH_2_OH), hydrogen peroxide (H_2_O_2_), N, N′-dimethylformamide (DMF), pH4.0 acetate-sodium acetate buffer, bovine serum albumin, RMPI-1640 medium, and fetal bovine serum without exosomes (Exofree-FBS) were obtained from Shanghai Titan Technology Co., LTD (Shanghai, China). CD63 aptamer was supplied by Sangon Biotech (Shanghai, China). Cell lines SGC-7901 and LO2 were purchased from Cell Bank affiliated with the Chinese Academy of Sciences.

### 2.2. The Preparation of MoS_2_-MIL-101(Fe)

MoS_2_ nanoflowers were synthesized by the hydrothermal method as previously reported [37]. In brief, 50 mg of (NH_4_)_2_MoS_4_ was dispersed in 30 mL of DMF under ultrasonic treatment for 15 min. Then, the solution was transferred into a Teflon liner and was kept at 200 °C for 10 h. After being cooled down, the raw product was washed with DMF and ethanol several times. After being dehydrated at 60 °C in a vacuum drier, MoS_2_ nanoflowers were purified and collected.

**Figure 1:**
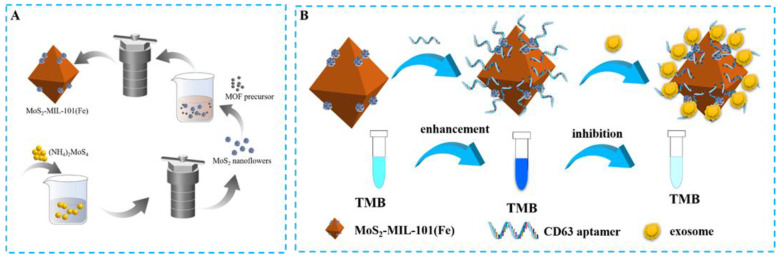
Schematic illustration of (**A**) the synthesis process of MoS_2_-MIL-101(Fe) and (**B**) the detection mechanism of the proposed method for exosomes.

MoS_2_-MIL-101(Fe) nanocomposites were synthesized via heat treatment of a mixture of FeCl_3_·6H_2_O, terephthalic acid, and MoS_2_ nanoflowers. In detail, under ultrasonic treatment, 20 mg of as-prepared MoS_2_ nanoflowers were dispersed in 15 mL of DMF to form a homogeneous solution. Then 0.206 g of terephthalic acid and 0.675 g of FeCl_3_·6H_2_O were dissolved in the abovementioned solution by continuous stirring. The mixture was transferred into a Teflon liner and was kept at 110 °C for 20 h. After being cooled down to room temperature, the raw product was separated, and washed by DMF and ethanol three times. Finally, after being dehydrated at 60 °C in a vacuum drier, MoS_2_-MIL-101(Fe) nanocomposites were collected (Figure 1A). In addition, MIL-101(Fe) nanocomposites were synthesized through the same processes described above except for the introduction of MoS_2_ nanoflowers [38].

### 2.3. Cell Culture and Exosomes Preparation

The SGC-7901 cells and LO2 cells were cultured in RMPI-1640 supplemented with 10% FBS at 37 °C in 5% CO_2_. After the cells proliferated to 80% confluence, the culture medium was replaced with RMPI-1640 supplemented with 10% Exofree-FBS. After 48 h incubation of exosome-free medium, the cell culture supernatant was collected to harvest tumor-derived exosomes, and the cells were passaged and cultured. Exosomes were isolated from the cell culture supernatant by the standard ultracentrifugation method with slight modifications [39]: (1) 1500× *g* centrifugation for 10 min to eliminate dead cells; (2) 1000× *g* for 20 min to eliminate cellular debris and the acquired supernatant was filtrated by a 0.22 μm filter; (3) 100,400× *g* for 4 h to precipitate exosomes.

### 2.4. Simulation of Peroxidase Activity in Nanocomposites

In the presence of a given concentration of H_2_O_2_ in a pH 4.0 HAc-NaAc buffer, the simulation of peroxidase activity in MoS_2_-MIL-101(Fe) nanocomposite was evaluated by introducing MoS_2_-MIL-101(Fe) hybrids to a TMB solution. The total volume of the reaction solution was set to 4 mL. The solution was composed of 1 mL of pH 4.0 HAc-NaAc buffer, 1 mL of MoS_2_-MIL-101(Fe) solution (50 mg/L), 1 mL of H_2_O_2_ solution (10 mM), and 1 mL of TMB solution (5 mM). Next, the reaction solution was incubated at 40 °C for 5 min and the absorbance was measured by an ultraviolet-visible (UV-vis) spectrophotometer.

Kinetic experiments of MoS_2_-MIL-101(Fe) hybrids were performed by measuring the initial rate of the reaction in the first 5 min, with one of the concentrations of H_2_O_2_ and TMB fixed, and the other varied. The H_2_O_2_ concentration was set to 4 mmol/L and the fixed TMB concentration was set to 2.5 mmol/L.

The kinetic parameters were fitted by the following equation: V_0_ = $V_{max}\frac{\left[S\right]}{K_{m}+[S]}$ . Here, K_m_ stands for Mi’s constant, [S] for substrate concentration, V_0_ for initial reaction rate, and V_max_ for maximum reaction rate. For each preset H_2_O_2_ and TMB concentrations, K_m_ and V_max_ were calculated by Hyperbola curve fit using the OriginPro 2019 (OriginLab Corporation, Northampton, MA, USA) after measuring V_0_. The hyperbolic function is also the Michaelis–Menten model in enzyme kinetics, and its formula is y = $\frac{k_{1}x}{k_{2}+x}$ corresponding to the Michaelis–Menten equation V_0_ = $V_{max}\frac{\left[S\right]}{K_{m}+[S]}$ . After being fitted, k_1_ is V_max_ and k_2_ is K_m_ [38].

### 2.5. Exosomes Detection

Ten microliters of CD63 aptamer (10 μM) and 200 μL MoS_2_-MIL-101(Fe) nanocomposites (100 mg/L) were mixed and blended by vortex. Then the different solutions with different concentrations of exosomes (10 μL) were added to the mixtures and were blended by vortex. After 30 min of incubation, 100 μL TMB (2.5 mM) solution and 100 μL H_2_O_2_ (4 mM) solution were added to the mixtures, and then HAc-NaAc buffer was added to fill up the volume of mixtures to 1000 μL. The mixtures were incubated at 40 °C for 5 min in the dark, and then the absorbances were measured by a UV–vis spectrophotometer with a 1.0 cm quartz cell.

## 3. Results and Discussion

### 3.1. Subsection

#### 3.1.1. The Principle and Feasibility of the Aptamer Sensor

**Figure 2:**
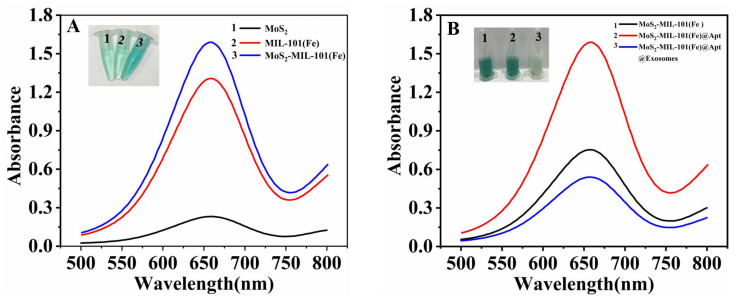
(**A**) Absorptions at 653 nm of different materials by UV-vis spectrophotometry, (1) MoS_2_; (2) MIL-101(Fe); (3) MoS_2_-MIL-101(Fe). (**B**) Absorptions at 653 nm of different materials by UV-vis spectrophotometry after the incubation of exosomes, (1) MoS_2_-MIL-101(Fe); (2) MoS_2_-MIL-101(Fe)@Apt; (3) MoS_2_-MIL-101(Fe)@Apt@Exosomes.

**Figure 3:**
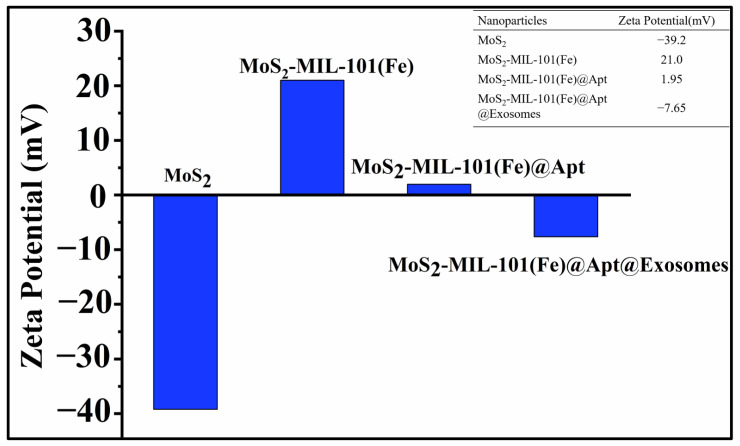
Zeta potential of different materials.

In order to synthesize hybrid nanozymes, nanoflower-like MoS_2_ materials were first synthesized by hydrothermal method. Then, the MoS_2_-MIL-101(Fe) nanocomposites were constructed on the basis of MOF precursor and MoS_2_ nanoflowers (Figure 1A). The design sketch of the MoS_2_-MIL-101(Fe) hybrid nanozyme-based aptamer sensor is shown in Figure 1B. The chromogenic reaction was introduced to evaluate and compare the peroxidase catalytic activities of the hybrid nanozyme, as well as its precursors. MoS_2_-MIL-101(Fe) nanocomposite was suggested to have a higher peroxidase activity in contrast to both MoS_2_ and MIL-101(Fe) (Figure 2A), which catalyzed TMB to transform from the colorless substrates to the deep blue substances in the presence of H_2_O_2_. Modified by CD63 aptamers, the peroxidase catalytic activity of the MoS_2_-MIL-101(Fe) was magnified, which is attributed to π-π stacking between single-stranded DNA and the substrate (Figure 2B). In detail, the bases of DNA aptamer facilitate the bindings between the substrates, especially through hydrogen bonding between DNA bases and the amino groups of TMB, as well as the nucleobase interacting between DNA bases and the benzene rings of TMB via π-π stacking, which led to the increased substrate affinity and further enhanced the catalytic activity of MoS_2_-MIL-101(Fe) [35,40]. In the presence of exosomes, the specific ligand-receptor recognition between CD63 aptamers and the CD63 proteins on the exterior of exosomes confined exosomes within the external surface of MoS_2_-MIL-101(Fe)@Aptamer and rescued the enhanced peroxidase catalytic activity of MoS_2_-MIL-101(Fe)@Aptamer, where MoS_2_-MIL-101(Fe)@Aptamer@Exosomes exhibited an even weaker peroxidase catalytic activity than MoS_2_-MIL-101(Fe) (Figure 2B). With various concentrations of exosomes, the hybrid nanozyme exhibited different degrees of peroxidase catalytic activities, so as to present different absorbances of the TMB colorimetric reaction, which could be obviously visualized and measured by UV–vis spectrometer. Therefore, the exosome-detection aptamer sensor was constructed based on the integration of CD63 aptamer and the hybrid nanozyme, which was verified with the following zeta potential measurements. As shown in Figure 3, the zeta potential of MoS_2_-MIL-101(Fe) is 21.0 mV, which shows that the composite material was positively charged. After incubating with the aptamer, the zeta potential of the composite material changed to 1.95 mV, which suggested that the CD63 aptamers were modified on the surface of MoS_2_-MIL-101(Fe) through electrostatic interaction. In addition, while the CD63 aptamers combined with the exosomes, the overall zeta potential of MoS2-MIL-101(Fe)@Aptamer@Exosomes turned to −7.65 mV, which continued to decline; the nanocomposite was negatively charged (Figure 3).

#### 3.1.2. The Characterization of MoS_2_-MIL-101(Fe) and Quantification of Exosomes

**Figure 4:**
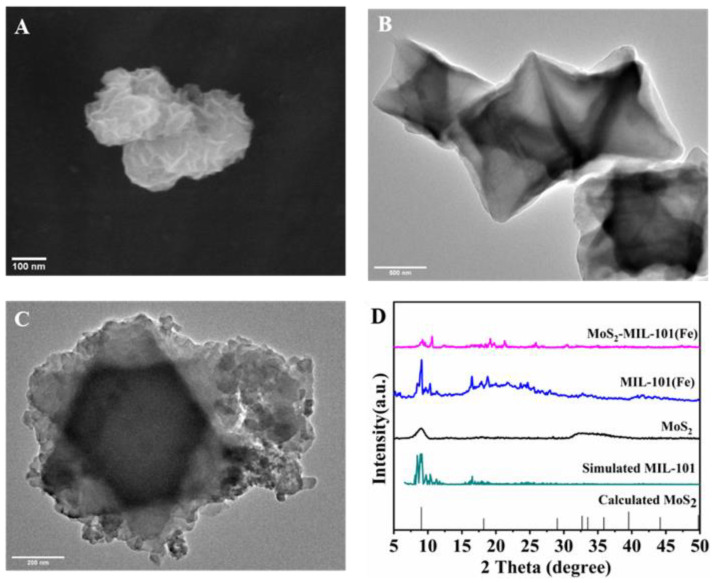
(**A**) The morphology of MoS_2_ revealed by SEM; (**B**) The morphology of MIL-101(Fe) revealed by TEM; (**C**) The morphology of MoS_2_-MIL-101(Fe) revealed by TEM; (**D**) The XRD pattern of MoS_2_-MIL-101(Fe) and its precursors.

The morphology of MoS_2_-MIL-101(Fe) nanocomposites and MoS_2_ was demonstrated by scanning electron microscopy (SEM) and transmission electron microscopy (TEM) (Figure 4). MoS_2_ presented a flower-like shape with a uniform size (ca. 150 nm) in diameter (Figure 4A), and MIL-101(Fe) displayed an octahedral nanostructure (Figure 4B). The successful preparation of the hybrid nanozyme was observed by TEM (Figure 4C) and further identified with X-ray diffraction (XRD) (Figure 4D), depicting a characteristic peak at 9.4° which is attributed to the (001) reflection of MoS_2_.

**Figure 5:**
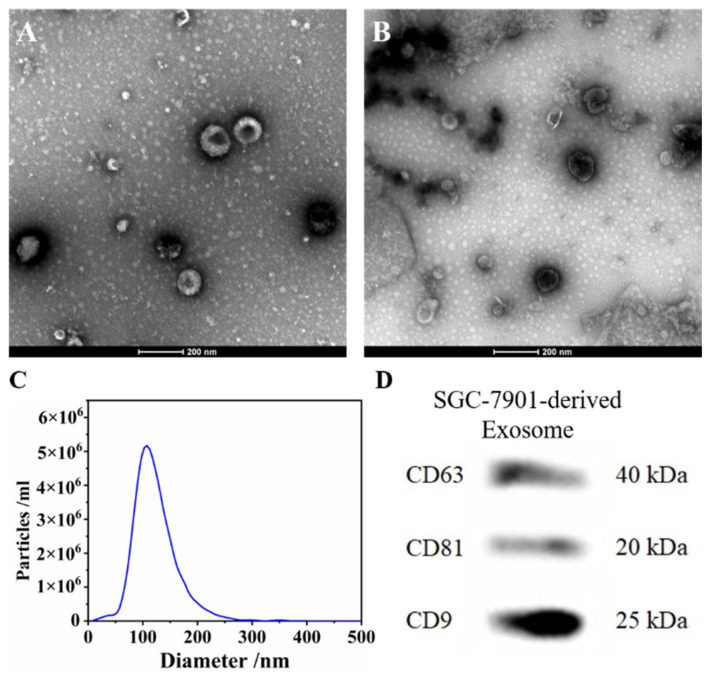
(**A**) The morphology of SGC-7901 cells-derived exosomes revealed by TEM; (**B**) The morphology of LO2 cells-derived exosomes revealed by TEM; (**C**) Exosome concentrations and particle sizes distribution of SGC-7901 cells; (**D**) The Western blots of SGC-7901 cells-derived exosomes.

Exosomes were isolated by ultracentrifugation from either human gastric cancer cell line SGC-7901 cells or normal human liver cell line LO2. The morphologies of the purified exosomes were demonstrated by TEM (Figure 5A,B), which showed an average diameter of 100 nm that coincided with the previous studies. The exosome counting was carried out by NTA, and the results were further used as the standard of exosomes. (Figure 5C). The expression of typical labeled proteins [41] (transmembrane proteins CD9, CD63 and CD81) of exosomes derived from SGC-7901 was directly verified by Western Blots (WB) (Figure 5D).

#### 3.1.3. Optimization of Experimental Conditions

The optimal experimental condition of the TMB reaction under MoS_2_-MIL-101(Fe)@Aptamer was then investigated, with the total reaction volume set to 4 mL. The activity of the nanozyme active site is affected by pH. Generally, the POD-like activity of the metal-based nanozyme is more efficient in an acidic environment while its catalase-like (CAT-like) activity is more efficient in an alkaline environment, where the transferred domination between POD- and CAT-like activities were driven through different reactant decomposition pathways at different pH [42]. The peroxidase catalytic activities of MoS_2_-MIL-101(Fe) at various pH conditions (from pH 2 to pH 8) were first investigated. The results showed that the catalytic activity was correlated with pH value, which presented the maximum activity at pH 4 (Figure S1A). Therefore, pH 4 was selected as the ideal pH value. Temperature also can regulate the catalytic activity of nanozymes. With increasing temperatures, the reactants’ thermal motions in the vicinity of active sites can be further activated, thus enhancing the catalytic activities of each individual active site. Therefore, the probability of molecular collisions between nanozymes and substrates is greatly proposed, thereby lifting the reaction rate [43]. Since enzymatic catalytic activity can be affected by temperature, the catalytic activities under different temperatures were also explored, which showed that the catalytic activity reaches to maximum at 40 °C (Figure S1B). Therefore, 40 °C was selected as the optimal temperature.

Then, the effect of different CD63 aptamer concentrations (from 2 μM to 20 μM) on the absorbances at 653 nm (A653) that reflect the concentrations of oxidized TMB was investigated. The A653 gradually increased as the CD63 aptamer concentration increased from 2 μM to 10 μM, achieved the highest value at 10 μM, and then gradually fell off when the concentration was above 10 μM (Figure S1C), which suggested that the optimal concentration of CD63 aptamer was 10μM. Similarly, the effects of different TMB concentrations, different H_2_O_2_ concentrations, and different MoS_2_-MIL-101(Fe) concentrations on A653 were monitored. It is reported [44] that the colorific reaction of benzidine under one-electron, or two-electron oxidation is blue or yellow, respectively, where the corresponding absorption peak is 653 nm or 450 nm. With increasing concentrations of TMB or hydrogen peroxide, the nanozyme-catalytic reaction rate, as well as the reaction rate of one-electron benzidine oxidation are lifted. Meanwhile, as a substrate, the product of one-electron benzidine oxidation also promotes two-electron oxidation, which causes the blue to yellow-green transformation of the solution system, leading to the decrease in the absorption peak at 653 nm. And it is consistent with the experimental phenomenon. The optimal conditions of these variables were shown as the followings: 2.5 mM TMB (Figure S1D), 4 mM H_2_O_2_ (Figure S1E), 20 μg/mL MoS_2_-MIL-101(Fe) (Figure S1F), which were chosen in the following experiments, respectively.

#### 3.1.4. The Kinetic Properties of MoS2-MIL-101(Fe) as a Peroxidase Simulator

**Figure 6:**
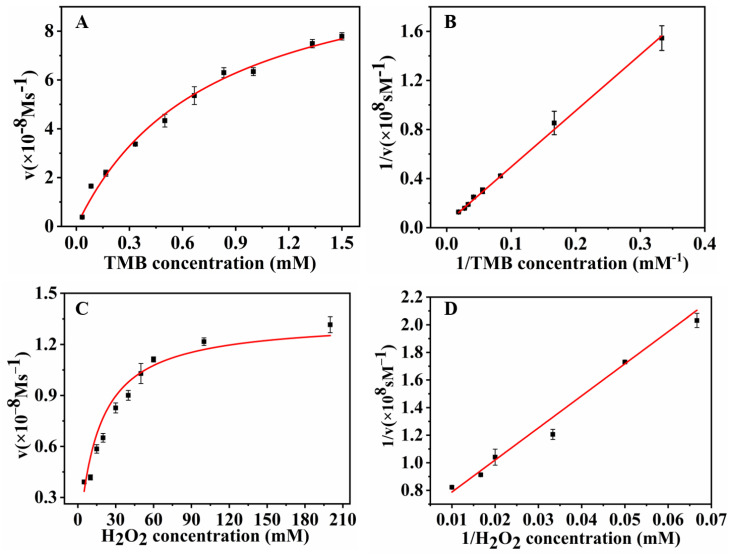
(**A**) Enzymatic reaction kinetics of the hybrid nanozyme on TMB; (**B**) The double reciprocal graph of enzymatic reaction kinetics of the hybrid nanozyme on TMB; (**C**) Enzymatic reaction kinetics of the hybrid nanozyme on H_2_O_2_; (**D**) The double reciprocal graph of enzymatic reaction kinetics of the hybrid nanozyme on H_2_O_2_.

Next, the steady-state kinetic experiment was applied to explore the kinetic properties of MoS_2-_MIL-101(Fe) as a peroxidase simulator, as well as its precursors, MIL-101(Fe) and MoS_2_. The apparent kinetic parameters, K_m_ and V_max_, were measured with one of the concentrations of H_2_O_2_ and TMB fixed, and the other varied. The hyperbolic kinetic curves of the hybrid nanozyme on TMB and H_2_O_2_ indicated that it followed the typical Michaelis–Menten pattern (Figure 6A,C) and possessed enzymatic kinetic properties. The lower apparent K_m_ value reflects the stronger affinity of the hybrid nanozyme to its substrates. The apparent K_m_ value of MoS_2_-MIL101(Fe) to TMB is 0.12 mM. In addition, the apparent K_m_ value of MoS_2_-MIL-101(Fe) to H_2_O_2_ is 0.015 mM, which was 29 times inferior to that of natural horseradish peroxidase. The results indicated that the hybrid nanozyme had advanced affinities to both TMB and H_2_O_2_. The double reciprocal graphs of Figure 6A and Figure 6C correspond to Figure 6B and Figure 6D, respectively.

#### 3.1.5. Analytical Performance in Determination of Exosomes

**Figure 7:**
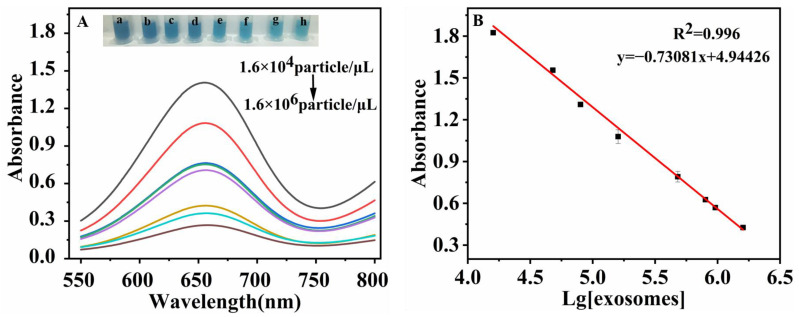
(**A**) Absorptions at 653 nm of the colorimetric aptasensor for detections on different exosome concentrations by UV-vis spectrophotometry (from a to h: exosome concentrations were 1.6 × 10^4^, 4.8 × 10^4^, 1.6 × 10^5^, 4.8 × 10^5^, 6.0 × 10^5^, 9.6 × 10^5^, 1.28 × 10^6^, 1.6 × 10^6^ particles/μL, respectively). The inset showed the corresponding colors of each concentration; (**B**) Linear relationship between A653 and the logarithm of exosome concentration.

Further, the absorbances at 653 nm (A653) of the TMB under diverse exosome concentrations were measured under the optimal conditions. As shown in Figure 7A, A653 decreased proportionally with the increase in exosome concentration. Correspondingly, color changes of the solutions with different exosome concentrations were obviously visible and comparable (a–h, Figure 7A). The linear range between A653 and the logarithm of exosome concentration is 1.6 × 10^4^~1.6 × 10^6^ particles/μL, where the correlation equation is y = −0.73081 × lg[c(exosomes)] + 4.94426, and the squared correlation coefficient is 0.996 (y reflects A653, and c(exosomes) reflects the exosome concentration, Figure 7B). As reported [45,46], a cancer cell can secrete more than 10^4^ vesicles in 24 h in contrast to a normal epithelial cell. In the clinical approach of exosome detection [47,48], our work could reach the level of detecting tumor-derived exosomes. Designed on the basis of the 3σ method (σ is the blank standard deviation), this aptamer sensor exhibited a promising limit of detection (LOD) of 3.37 × 10^3^ particles/μL, which is lower than that of other methods reported and is attributed to the high POD-like catalytic activity of the hybrid nanozyme (Table 1) [49,50,51,52,53,54,55,56].

#### 3.1.6. The Selectivity, Reproducibility, and Stability of the Aptamer Sensor

**Figure 8:**
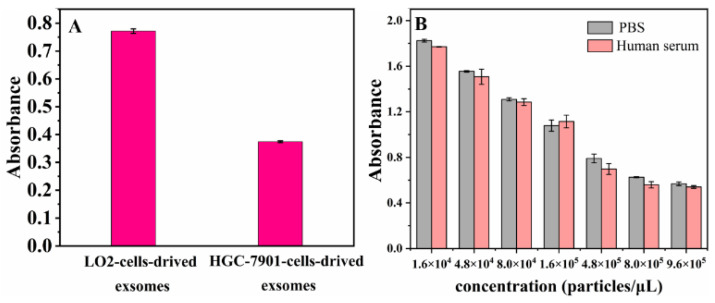
(**A**) The histogram of selectivity of MoS_2_-MIL-101(Fe) nanozyme-based aptasensor. Each column exhibited the average value of three independent data with an error bar presented; (**B**) Comparison of absorbances between PBS and human serum with the same concentration of exosomes. Each column exhibited the average value of three independent data with an error bar presented.

The specificity was a pivotal issue for developing a novel exosome-detection aptamer sensor. Heterogeneous exosomes with different CD63 protein expressions were adopted to verify the specificity of this aptamer sensor. As previously reported [36], the CD63 expressions of exosomes derived from human gastric cancer cell line SGC-7901 cells were lower than those derived from normal human liver cell line LO2. As shown in Figure 8A, the stronger signals were observed in the group of CD63-low SGC-7901 cells-derived exosomes, while the much weaker signals were observed in the group of CD63-high LO2 cells-derived exosomes with the same concentration of exosomes. In accordance with the previous studies, the results demonstrated that this aptamer sensor had a good selectivity on various exosomes based on the specificity of the CD63 aptamer.

The reproducibility of this aptasensor has also been explored. As shown in Figure S2, the exosome detection effectiveness of the aptasensor after 30-day storage at 4 °C exhibited a good similarity with that of synthesized on the first day as its RSD values still presented consistencies less than 5%, which indicated favorable reproducibility, stability, and the shelf-life of this aptasensor.

#### 3.1.7. Detection of Exosomes in Human Serum Sample

To explore the applicability of this hybrid nanozyme-based aptamer sensor on a clinical approach, SGC-7901-derived exosomes were added to human serum to establish artificial samples for detection. The results showed that the calculated recoveries ranged from 95% to 103% (Table S1). As shown in Figure 8B, exosome detections of the artificial samples presented results consistent with those of PBS samples with the same concentration of exosomes added. These results clearly indicated that exosome detection based on this aptasensor is accurate and suitable for detection in complex systems, providing an important tool for exosome detection in clinical applications.

## 4. Conclusions

In conclusion, a novel, sensitive, visible and simple approach to exosome detection is developed by means of the MoS_2_-MIL-101(Fe) hybrid nanozyme-based aptasensor. Excellent linearity was obtained for exosome detection within the extent of 1.6 × 10^4^ to 1.6 × 10^6^ particles/μL, as well as a LOD of 3.37 × 10^3^ particles/μL was achieved. The aptamer sensor possesses equal applicability in complex biological samples in clinical approach, which exhibited a cutting-edge economic efficiency and accessibility that can be potentially applied for portable exosome-detection devices. Nonetheless, in this study, there are limitations that need to be pointed out that the unsolved defects of nanozyme, such as the relatively low substrate selectivity and catalytic efficiency, still impede the further enhancement of the sensitivity of this aptasensor. In addition, the single CD63 DNA aptamer design of this aptasensor also hinders the further improvement of its exosome specificity.

## Figures and Tables

**Table 1:** Comparisons with various reported strategies for exosome detection.

Method	Linear Range (Particles/μL)	LOD (Particles/μL)	Reference
Electrochemical (Paper-based Device)	2.47 × 10^5^–2.47 × 10^6^	7.1 × 10^5^	[49]
Electrochemical (Au NPs)	9 × 10^6^–1.4 × 10^7^	4.5 × 10^6^	[50]
Fluorescent (CD63-MBs)	1.66 × 10^3^–1.66 × 10^6^	4.8 × 10^2^	[51]
Fluorescent (G-quadruplex)	5.0 × 10^5^–5.0 × 10^7^	3.4 × 10^5^	[52]
Fluorescence (CuO NPs)	7.5 × 10^4^–1.5 × 10^7^	4.8 × 10^4^	[53]
Colorimetric (Carbon Nanotubes)	10^6^–10^8^	3.94× 10^4^	[54]
Colorimetric (Fe_3_O_4_)	4.0 × 10^5^–6.0 × 10^7^	3.58 × 10^3^	[55]
Colorimetric (Fe-MIL-88)	1.1 × 10^5^–2.2 × 10^7^	5.2 × 10^4^	[56]
Colorimetric (CuCo_2_O_4_)	5.6 × 10^4^–8.9 × 10^5^	4.5 × 10^3^	[57]
Colorimetric (ZnO)	2.2 × 10^5^–2.4 × 10^7^	2.2 × 10^4^	[58]
Colorimetric (MoS_2_-MIL-101(Fe))	1.6 × 10^4^–1.6 × 10^6^	3.37 × 10^3^	This work

## Data Availability

Data are available upon reasonable request from the corresponding authors.

## Supplementary Materials

The following supporting information can be downloaded at: https://www.mdpi.com/article/10.3390/bios13080800/s1, Figure S1: Influences of (A) pH, (B) temperature, (C) aptamer concentration, (D) TMB concentration, (E) H2O2 concentration, (F) MoS2-MIL-101(Fe) concentration for the MoS2-MIL-101(Fe) hybrid nanozyme-based aptasensor; Figure S2: Comparison of absorbances between measurement at first-day synthesis and measurement after 30-day room-temperature storage with the same concentration of exosomes. Each column exhibited the average value of three independent data with an error bar presented; Table S1: Detection of SGC-7901-derived exosomes in human serum.
