# Correction: Li et al. Molybdenum Disulfide-Integrated Iron Organic Framework Hybrid Nanozyme-Based Aptasensor for Colorimetric Detection of Exosomes. *Biosensors* 2023, *13*, 800

**DOI:** 10.3390/bios15090609

**Published:** 2025-09-16

**Authors:** Chao Li, Zichao Guo, Sisi Pu, Chaohui Zhou, Xi Cheng, Ren Zhao, Nengqin Jia

**Affiliations:** 1 The Education Ministry Key Lab of Resource Chemistry, Joint International Research Laboratory of Resource Chemistry, Ministry of Education, Shanghai Frontiers Science Center of Biomimetic Catalysis and Shanghai Key Laboratory of Rare Earth Functional Materials, College of Chemistry and Materials Science, Shanghai Normal University, Shanghai 200234, China; 1000495334@smail.shnu.edu.cn (C.L.); sisi.pu@outlook.com (S.P.); chaohuizhou@shnu.edu.cn (C.Z.); 2 Department of General Surgery, Ruijin Hospital, Shanghai Jiao Tong University School of Medicine, Shanghai 200025, China; guozichao@sjtu.edu.cn

## Error in Figure

In the original publication [1], there was a mistake in Figure 5D as published, The cell line HGC-7901 should be modified to SGC-7901. The corrected Figure 5D appears below. The authors state that the scientific conclusions are unaffected. 


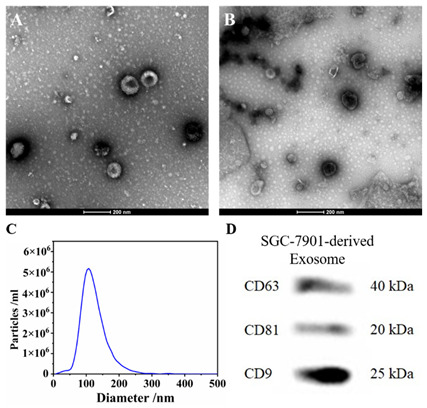


## Text Correction

There were errors in the original publication, including The cell line HGC-7901 should be modified to SGC-7901. A correction has been made to Section 2.1, Section 2.3, Section 3.1.2, Caption of Figure 5, Section 3.1.6, Section 3.1.7, and Section Supplementary Materials:

Cell lines SGC-7901 and LO2 were purchased from Cell Bank affiliated with the Chinese Academy of Sciences.

The SGC-7901 cells and LO2 cells were cultured in RMPI-1640 supplemented with 10% FBS at 37 °C in 5% CO_2_.

Exosomes were isolated by ultracentrifugation from either human gastric cancer cell line SGC-7901 cells or normal human liver cell line LO2.

The expression of typical labeled proteins [41] (transmembrane proteins CD9, CD63 and CD81) of exosomes derived from SGC-7901 was directly verified by Western Blots (WB) (Figure 5D).

**Figure 5.** (**A**) The morphology of SGC-7901 cells-derived exosomes revealed by TEM; (**B**) The morphology of LO2 cells-derived exosomes revealed by TEM; (**C**) Exosome concentrations and particle sizes distribution of SGC-7901 cells; (**D**) The Western blots of SGC-7901 cells-derived exosomes.

As previously reported [36], the CD63 expressions of exosomes derived from human gastric cancer cell line SGC-7901 cells were lower than those derived from normal human liver cell line LO2. As shown in Figure 8A, the stronger signals were observed in the group of CD63-low SGC-7901 cells-derived exosomes, while the much weaker signals were observed in the group of CD63-high LO2 cells-derived exosomes with the same concentration of exosomes.

To explore the applicability of this hybrid nanozyme-based aptamer sensor on a clinical approach, SGC-7901-derived exosomes were added to human serum to establish artificial samples for detection.

Table S1: Detection of SGC-7901-derived exosomes in human serum.

The authors state that the scientific conclusions are unaffected. This correction was approved by the Academic Editor. The original publication has also been updated.
