# Supplementary material for: Molybdenum Disulfide-Integrated Iron Organic Framework Hybrid Nanozyme-Based Aptasensor for Colorimetric Detection of Exosomes
Source: Biosensors (Basel). 2023 Aug 9;13(8):800. doi: 10.3390/bios13080800 (PMC10452346; doi:10.3390/bios13080800)
Supplement: Supplementary file 1 [file biosensors-13-00800-s001.zip › biosensors-2501169-Supplementary Materials.pdf]

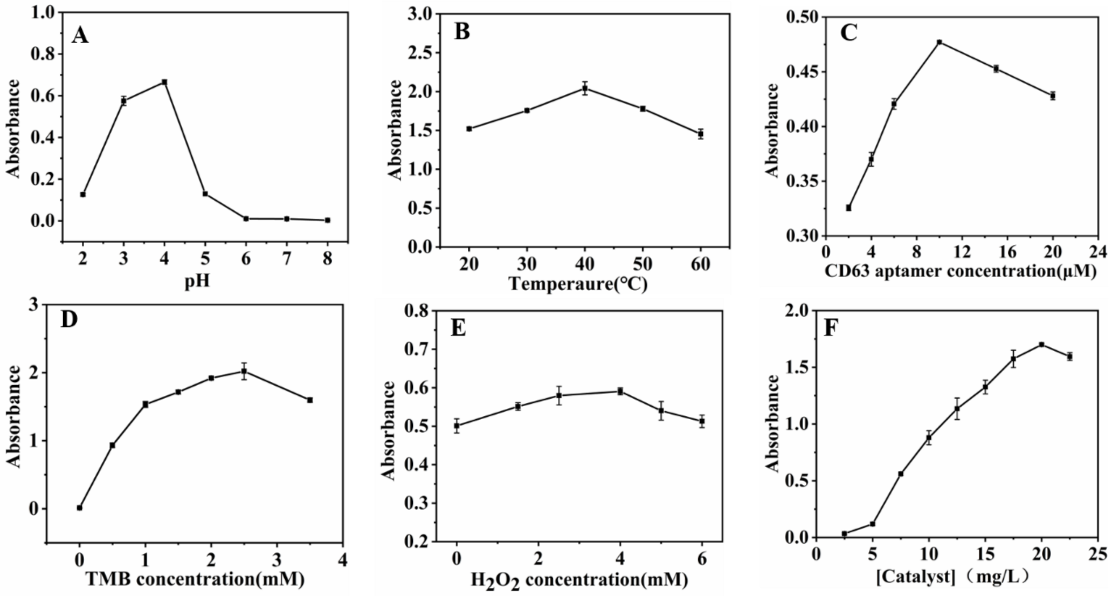

Figure S1. Influences of (**A**) pH, (**B**) temperature, (**C**) aptamer concentration, (**D**) TMB concentration, (**E**) H_2O_2 concentration, (**F**) MoS_2-MIL-101(Fe) concentration for the MoS_2-MIL-101(Fe) hybrid nanozyme-based aptasensor.

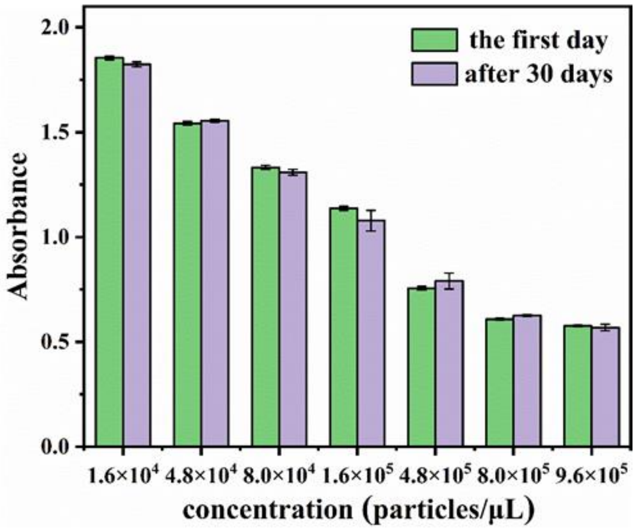

Figure S2. Comparison of absorbances between measurement at first-day synthesis and measurement after 30-day storage at 4 °C with the same concentration of exosomes. Each column exhibited the average value of three independent data with an error bar presented.

Table S1. Detection of SGC-7901-derived exosomes in human serum.

Sample Number	Added (Particles/μL)	Found (Particles/μL)	Recovery (%)	RSD (%) (n = 3)
1	1.60×10^4	1.54×10^4	103	0.1
2	4.80×10^4	4.65×10^4	97	4.4
3	8.00×10^4	7.86×10^4	98	2.3
4	1.60×10^5	1.65×10^5	103	5.0
5	9.60×10^5	9.12×10^5	95	2.2
